# Modulation of Ion Transport to Restore Airway Hydration in Cystic Fibrosis

**DOI:** 10.3390/genes12030453

**Published:** 2021-03-22

**Authors:** James A. Reihill, Lisa E. J. Douglas, S. Lorraine Martin

**Affiliations:** School of Pharmacy, Queen’s University Belfast, Belfast BT9 7BL, UK; L.Douglas@qub.ac.uk (L.E.J.D.); l.martin@qub.ac.uk (S.L.M.)

**Keywords:** cystic fibrosis, ion channel, cystic fibrosis transmembrane conductance regulator (CFTR), epithelial sodium channel (ENaC), transmembrane member 16A (TMEM16A), big potassium (BK), airway hydration

## Abstract

Cystic fibrosis (CF) is a life-limiting genetic disorder caused by loss-of-function mutations in the gene which codes for the CF transmembrane conductance regulator (CFTR) Cl^−^ channel. Loss of Cl^−^ secretion across the apical membrane of airway lining epithelial cells results in dehydration of the airway surface liquid (ASL) layer which impairs mucociliary clearance (MCC), and as a consequence promotes bacterial infection and inflammation of the airways. Interventions that restore airway hydration are known to improve MCC. Here we review the ion channels present at the luminal surface of airway epithelial cells that may be targeted to improve airway hydration and MCC in CF airways.

## 1. Introduction

The airway surface liquid (ASL), comprising a thin layer of fluid that bathes cilia (the periciliary layer; PCL) and an overlying mucus layer, is continuously propelled by a coordinated cilia beat from the distal to proximal airways. This mucociliary escalator is a critical component of innate immune defence, protecting the lungs from inhaled agents including respiratory pathogens. In cystic fibrosis (CF), the loss of the CF transmembrane conductance regulator (CFTR) has devastating consequences for ASL homeostasis. A substantially reduced capacity for Cl^−^ secretion from epithelial cells to the luminal surface and a concomitant increase in Na^+^ absorption via the epithelial sodium channel (ENaC) results in increased cytosolic NaCl levels that create an osmotic driver to remove water from the ASL [1]. Dehydration of the airway surfaces and hyperconcentration of mucus results in PCL collapse and compromised mucociliary clearance (MCC). The ensuing mucus accumulation is regarded as a major contributor to CF lung disease pathogenesis [2].

**Figure 1:**
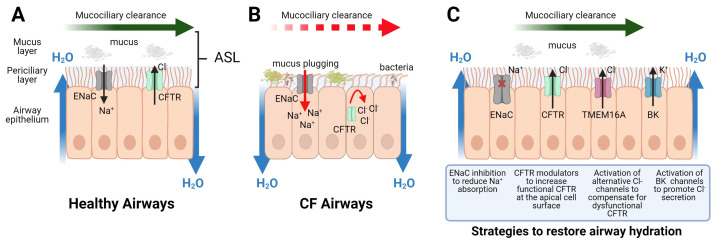
Modulation of ion transport in cystic fibrosis (CF) airways to restore the airway surface liquid. The airway surface liquid (ASL) comprises the watery periciliary fluid and overlying mucus layer that traps inhaled particles for removal from the lungs by mucociliary clearance (MCC). (**A**) In healthy airways, CF transmembrane conductance regulator (CFTR) and epithelial sodium channel (ENaC) work in concert to appropriately control the movement of Cl^−^ and Na^+^, respectively, maintaining an optimal ASL and effective mucociliary clearance. (**B**) In CF airways, the loss of CFTR-mediated Cl^−^ secretion and ENaC hyperabsorption results in the collapse of the PCL which restricts cilia function and MCC, resulting in mucus plugging and opportunity for bacterial colonisation. (**C**) A number of apically expressed ion channels may be targeted in CF airways to restore airway hydration. CFTR modulators (potentiator and/or corrector(s)) can partially restore CFTR-mediated Cl^−^ secretion whereas inhibition of ENaC has potential to improve airway hydration, alone or in combination with CFTR modulator therapy. Additional strategies which include activation of TMEM16A, a CFTR-independent route to increase Cl^−^ secretion, and activation of BK (big potassium) channels, offer further opportunity to improve airway hydration.

Small molecule CFTR modulator compounds have recently been approved for clinical use. This ground-breaking work highlights pharmacological tools that modulate ion channel function and improve airway hydration as a valid therapeutic approach. These medicines, however, are not curative and a number of challenges remain. Targeting of alternative ion channel activities that can compensate for CFTR dysfunction and promote airway hydration, therefore, offers further opportunity for the development of drugs that may be used as monotherapies and/or in combination with existing CFTR modulators. This review provides an overview of the progress made with CFTR modulator therapies, the remaining challenges to be overcome and how modulation of alternative ion channels may advance the treatment of CF lung disease. The various approaches are summarised in Figure 1.

## 2. Ion Channel Targets

### 2.1. CFTR Modulators

Historically treatments for CF have been symptom-based, tackling mucus obstruction, infection and inflammation, which has incrementally improved life expectancy and quality of life for people with CF (PWCF). However, we have now entered a new era where small-molecules that rescue mutant-CFTR channel function are successfully being used in the clinic. The first, ivacaftor (VX-770; Kalydeco^®^, Vertex Pharmaceuticals, Boston, MA, USA), a potentiator drug that improves the open probability of mutant-CFTR where there is defective gating (e.g., Class III residual function mutations such as G551D) [3], was shown to translate to improved clinical outcomes to include an increase in lung function (percent predicted forced expiratory volume in one second (ppFEV_1_) ~11%) [4]. Ivacaftor however, is only suitable for a small subgroup of PWCF and does not provide benefit for the majority of individuals (~80%) who harbour the most common mutation, a deletion of a phenylalanine residue at position 508 on CFTR (Phe508del) [5]. The Phe508del mutation results in CFTR protein mis-folding that is identified by endoplasmic reticulum (ER) quality control mechanisms and targeted for degradation. This limits the amount of Phe508del-CFTR that traffics to the apical surface to a very minor fraction. The Phe508del-mutant CFTR that does successfully arrive to the apical cell surface also exhibits defective channel gating and protein stability. To circumvent these issues, it has been necessary to employ combination treatments involving a potentiator drug (ivacaftor) with one or more corrector compounds that rescue the folding, processing and trafficking of mutant CFTR. This has not been straightforward with potentiators shown to destabilise mutant CFTR in the presence of chronic corrector (lumacaftor; VX-809) administration [6,7], which may explain the relatively modest clinical benefits (ppFEV1 improvements of 3–4%) observed when potentiator/corrector dual therapies were examined in patient homozygous for Phe508del [8,9,10]. Efforts to improve CFTR correction have led to a triple combination therapy, known as Trikafta^®^ in the United States and under the brand name Kaftrio^®^ in Europe. This triple combination therapy (Trikafta/Kaftrio) consists of ivacaftor and two corrector drugs, tezacaftor (VX-661) and elexacaftor (VX-445), that work in an additive fashion to correct CFTR mis-folding and have been shown to significantly improve lung function (11–14% in ppFEV1) in PWCF, including both homozygous and heterozygous forms for Phe508del [11,12].

New classes of CFTR modulators, namely CFTR amplifiers, stabilisers and read-through agents, are under pre-clinical and clinical investigation and show potential for further advancement of CF pharmacotherapy. Stabilisers are aimed at increasing the residence time of CFTR at the plasma membrane and as such may possibly complement other CFTR modulator therapies, such as potentiators and correctors. Cavosonstat (N91115), developed by Nivalis Therapeutics (San Francisco, CA, USA), stabilises CFTR at the cell surface downstream of Snitroglutathione reductase inhibition [13]. Unfortunately, in Phase 2 clinical studies when cavosonstat was added in combination with Ivacaftor (NCT02724527) or with ivacaftor/lumacaftor (NCT02589236), there was no demonstrable improvement in lung function, thus further development of this compound has ceased. Amplifier agents increase the amount of mutant CFTR mRNA and subsequently the levels of CFTR protein, therefore whilst the function of the defective protein is not corrected directly, there is an increased amount of substrate on which other CFTR modulators can exert their effect. Neosolicaftor (PTI-428, Proteostasis Therapeutics) identified through a phenotypic high-throughput screen [14], was shown in pre-clinical studies to augment the actions of ivacaftor/lumacaftor (Orkambi^®^) [15]. A phase 2 clinical study assessing neosolicaftor in combination with ivacaftor/lumacaftor (NCT03591094) found increased CFTR protein expression in nasal mucosa but no significant impact on lung function. However, Proteostasis Therapeutics have recently announced that in a phase 1/2 clinical study where neosolicaftor was tested in combination with the CFTR modulators posenacaftor (PTI-801) and dirocaftor (PTI-808) (NCT03500263), there was an ~8% increase in ppFEV_1_ in CF adults homozygous for F508del. A phase 3 clinical study to advance the development of this triple combination therapy is intended. Read-through agents provide a mechanism to overcome nonsense mutations where there is the introduction of a premature termination codon (PTC), resulting in a truncated and defective CFTR protein. Approximately 10% of PWCF carry a PTC mutation (e.g., *G542X* and *W1282X*). ELX-02 (NB124) developed by Eloxx Pharmaceuticals (Waltham, MA, USA) has been shown to restore CFTR function in pre-clinical models examining the compound as read-through agent for CF caused by the G542X mutation [16,17]. Phase 2 clinical studies (NCT04126473 and NCT04135495) to test ascending doses of ELX-02 are underway for PWCF who have at least one copy of the G542X CFTR mutation.

### 2.2. Approved CFTR Modulator Therapies—Current Challenges

CFTR modulator therapy has improved the health and lives of many PWCF, yet challenges remain. Those who receive CFTR modulator therapy continue to experience pulmonary exacerbations, albeit at a reduced rate, and fail to eradicate bacteria from the airways over time [18]. It is evident that significant patient-to-patient variability exists concerning response to treatment, even amongst individuals that carry the same CFTR mutation. This may be due to a variety of factors, including the presence of gene modifiers [19] and the type of lung infection present [20,21]. Whilst Trikafta/Kaftrio is expected to be suitable for ~90% of PWCF, this still leaves a substantial subgroup of individuals that cannot benefit from this type of therapy due to an unsuitable CFTR mutation. In addition, these drugs are expensive and not universally available widening the pool of PWCF who are unable to access these treatments. Finally, the long-term safety of these drugs remains unknown. As such, even in the era of CFTR modulator therapy, it is imperative to develop new treatments for CF.

## 3. TMEM16A

The discovery that triphosphate nucleotides (e.g., adenosine-5′ triphosphate (ATP) or uridine-5′-triphosphate (UTP)) stimulate a calcium-activated Cl^−^ channel (CaCC), now known to be TMEM16A [22], in human airway epithelial cells has raised the possibility of a surrogate route for anion secretion independent of patient genotype [23]. Denufosol, a UTP analogue more resistant to degradation than native triphosphate nucleotides, was subsequently tested as an inhaled therapy for CF but failed to demonstrate clinical benefit [24]. These disappointing results may, at least in part, be due to desensitisation of the PY2Y receptor which prevents a sustained hydration of the airways [25]. Based on this, bypassing the purinergic signalling pathway and directly targeting TMEM16A has merit. In 2020, Enterprise Therapeutics (Brighton, UK) reported a novel TMEM16A potentiator compound (ETX001) (subsequently acquired by Roche and named ETD002), that works independently of calcium signalling to increase epithelial anion secretion and airway hydration in vitro and mucociliary clearance in a sheep model [26]. ETD002 is currently in phase I clinical trials (NCT04488705). Whilst a number of studies suggest TMEM16A activation may result in deleterious smooth muscle contraction and mucus secretion [27,28,29], potentiation of the channel mediated by ETX001 did not affect goblet cell numbers nor mucin release from differentiated airway epithelial cells or the contraction or relaxation of isolated human bronchi or pulmonary arteries [30].

## 4. ENaC

ENaC is a heterotrimeric channel consisting of αβγ subunits that is expressed at the apical surface of epithelial cells in a variety of tissues, including the airways. ENaC represents the rate-limiting step for reabsorption of sodium which drives paracellular movement of Cl^−^ and water. In CF airways where CFTR function is lost, ENaC activity not only remains intact but is elevated, promoting airway dehydration [31]. Indeed, a transgenic mouse model where ENaC is overexpressed results in a CF-like lung disease phenotype [32]. ENaC activity is regulated by several factors which affect either the open probability (P_o_) or the number of channels at the cell surface (*n*). P_o_ is dramatically increased via proteolytic cleavage of the α and γ subunits with conductance for Na^+^ otherwise found to be low. Several proteases have been shown to activate ENaC, including furin which processes the α and γ subunits as it passes through the biosynthetic pathway, as well as by extracellular channel activating proteases (CAPs) such as prostasin and neutrophil elastase which are present at elevated levels in the CF airways [33,34,35]. Notably, WT-CFTR, but not Phe508del CFTR, has been shown to protect ENaC from proteolytic activation [36]. Once activated, channel activity is reduced by removal of ENaC from the cell surface by endocytosis (i.e., a reduction in n). Based on these ENaC regulatory mechanisms strategies to normalise channel activity include direct channel blockers, inhibition of CAP activities or by increased removal of the channel from the cell surface.

### 4.1. Direct ENaC Blockers

The classic ENaC channel blocker amiloride is a potassium-sparing diuretic developed by Merck Pharmaceuticals in the 1960s. Amiloride was examined as an inhalation therapy to improve MCC in PWCF but failed to demonstrate clinical benefit due to insufficient potency and rapid clearance from the lungs [37]. Subsequently, several ENaC antagonists with improved potency and duration of action that enhanced airway hydration and mucociliary clearance in pre-clinical models have been examined in clinical studies, but unfortunately have failed to meet their primary outcome measures. These include P-1037/VX-371 (developed by Parion Science and progressed in collaboration with Vertex Pharmaceuticals) (NCT02709109) and AZD5634 (AstraZeneca) (NCT02950805). Currently BI 1265162 from Boehringer Ingelheim (Ingelheim, Germany) is the sole ENaC channel blocker in clinical development with phase 2 studies ongoing (NCT04059094). During pre-clinical assessment, BI 1265162 has been shown to inhibit Na^+^ transport in vitro, which correlates with observed increased mucociliary clearance rates in a sheep model [38].

### 4.2. Protease Inhibitors

A number of extracellular serine and metalloproteases have been shown to activate ENaC, thus priming protease inhibitor-based strategies to silence ENaC. Camostat, a broad spectrum inhibitor of trypsin-like proteases including the CAPs prostasin and matriptase, was shown to attenuate ENaC in guinea pig trachea and enhanced MCC in sheep for at least 5 h after inhaled dosing [39]. In the nasal potential difference (PD) assay, amiloride-sensitive PD, indicative of ENaC activity, was significantly reduced with camostat administration [40]. As ENaC is cleaved by a number of extracellular proteases, including those present in airway epithelium but also activities released from immune cells and pathogens makes it somewhat difficult to target the full range. Indeed, trypsin-like protease inhibitor-mediated inhibition of ENaC is reversed by the application of neutrophil elastase [34], which is present in significant quantities in the CF lung. A novel compound QUB-TL1, that inhibits trypsin-like CAPs similar to camostat and that additionally targets furin, has been shown to elicit a degree of protection from subsequent activation by extracellular proteases, including neutrophil elastase [34]. As furin-mediated processing of ENaC α/γ subunits is necessary for further downstream activation by CAPs this protease represents an intriguing therapeutic target, however a potent and selective drug-like furin inhibitor will be required.

### 4.3. Reduction of Surface ENaC Expression

SPLUNC1 is a secreted protein present in the airways that has been shown to regulate ENaC [41] via an 18 amino acid domain on the protein’s N-terminus [42]. An optimised peptide (SPX-101) based on this region was shown to internalise ENaC subunits, thus increasing airway hydration and MCC in CF human bronchial epithelial cells, and to reduce inflammation and improve survival of βENaC overexpressing mice as well as improving mucus clearance rates in a sheep model [43]. Early clinical studies (NCT03229252) found that SPX-101 was well-tolerated however, despite positive interim results evaluation of the full dataset revealed no significant improvement in lung function, thus the programme was discontinued [44].

### 4.4. Additional Approaches to Target ENaC

Antisense oligonucleotides (ASOs) enable the selective targeting of genes of interest. ASOs that target ENaC mRNA have been shown to improve lung disease in murine CF-like models [45]. Presently an ASO directed towards *SCNN1A (IONIS-ENAC-2.5_RX_)* (Ionis Pharmaceuticals, Carlsbad, CA, USA) to reduce lung ENaC expression is undergoing a phase 1/2a study in healthy volunteers and adults with CF (NCT03647228).

## 5. BK Channels

Large-conductance, calcium-activated and voltage-gated K^+^ (BK) channels play a role in excitable and non-excitable cells in a variety of tissues throughout the human body. BK channels, apically expressed at the surface of airway epithelial cells, influence airway hydration and mucociliary clearance by providing an electrochemical gradient for Cl^−^ secretion [46]. It has been demonstrated that compromised BK channel function can occur in response to inflammation [47]. The angiotensin receptor blocker, losartan (that possesses anti-inflammatory properties) has been shown to rescue BK channels and improve airway hydration in primary CF airway epithelial cells and mucociliary clearance in a sheep model [48]. A clinical trial (NCT03206788) to examine losartan and inflammation in CF was terminated due to slow enrolment and the approval of Trikafta/Kaftrio.

## 6. Summary

The successful development of CFTR modulator therapies provides proof-of-concept that pharmacological compounds can address the basic defect in CF and deliver clinical benefit to PWCF. Unfortunately, these treatments are not curative, nor are they available or suitable for all PWCF. Whilst efforts to further improve the levels of CFTR correction will undoubtedly continue, the targeting of alternative ion channels to improve airway hydration and mucociliary clearance represents a promising complementary strategy that would have the added benefit of working in a mutation-agnostic fashion, which could have broad benefit across the CF population as a whole.
